# The burden of disease due to COVID-19 in Sweden 2020–2021: A disability-adjusted life years (DALYs) study

**DOI:** 10.1177/14034948231160616

**Published:** 2023-03-20

**Authors:** Jad Shedrawy, Patricia Ernst, Knut Lönnroth, Fredrik Nyberg

**Affiliations:** 1 Department of Global Public Health, Karolinska Institutet, Sweden; 2 School of Public Health and Community Medicine, Institute of Medicine, Sahlgrenska Academy, University of Gothenburg, Sweden; 3 Centre for Epidemiology and Community Medicine, Stockholm County Council, Sweden

**Keywords:** COVID-19, DALYs, mortality, disability, Sweden, disease burden

## Abstract

**Background:**

The burden of COVID-19 disease can be measured in terms of disability-adjusted life years (DALYs), which is composed of two components: the years of life lost through premature death (YLL) and the number of years lived with disability (YLD), adjusted for level of disability. This study measured DALYs due to COVID-19 in Sweden and compared it to the burden of other diseases.

**Methods:**

The methodology used in the calculation of DALYs was based on the Global Burden of Disease guidelines. The number of patients diagnosed with mild/moderate, severe or critical COVID-19 and/or post-COVID-19 condition between March 2020 and October 2021 was extracted from national registries and used for YLD calculations. In addition, the numbers of death due to COVID-19 in different age groups were used for the YLL calculation.

**Results:**

During the study period, 152,877 DALYs were lost to COVID-19 in Sweden, 99.3% of which was attributed to YLL. Loss of DALYs occurred mainly among the elderly, with 66.8% of DALYs attributed to individuals >70 years old. Compared to other diseases, the burden of COVID-19 in 2020 ranked as the eighth leading cause of DALY lost.

**Conclusions:**

**Similar to other countries, the burden of COVID-19 in Sweden was concentrated mainly among the elderly, who contributed most of the DALY lost due to premature mortality. Yet, DALY loss remained lower for COVID-19 than for several other diseases. The contribution of YLD to DALYs lost was minimal. However empirical data on the occurrence and disability of post-COVID-19 condition are scarce, and YLD may therefore be underestimated.**

## Introduction

At the end of 2019, severe acute respiratory syndrome coronavirus 2 (SARS-CoV-2), which causes the novel coronavirus disease 2019 (COVID-19), was first reported in Wuhan, China [1]. It was declared a public health emergency of international concern by the World Health Organization in January 2020 [2] and quickly evolved into a pandemic. Nearly 604 million COVID-19 cases and more than 6.4 million deaths had been reported worldwide as of the beginning of September 2022 [3]. However, officially reported COVID-19 cases and death numbers are considered an underestimation in many countries [4].

Most countries worldwide were challenged by the COVID-19 pandemic, including Sweden. The first case in the country was detected on 24 January 2020 in a woman who had recently returned from China [5]. The number of cases then increased during different pandemic waves, leading to more than 2,500,000 cases and around 20,000 deaths, corresponding to a cumulative rate of 1975 deaths/million by September 2022, as reported by the Public Health Agency [6].

To study the impact of COVID-19 on population health and contextualise this impact compared to other diseases or across countries, burden of disease assessments have been conducted in different countries, estimating disability-adjusted life years (DALYs) lost due to COVID-19. DALY is a common outcome that allows for estimating the impact of diseases, such as COVID-19, in terms of both morbidity and mortality combined [7]. It has two main components: the years of life lost through premature mortality (YLL) and the number of years lived with disability, weighted for the level of disability (YLD). YLDs reflect years lived with less-than-ideal health due to disability or morbidity, thus considering both the severity and duration of the disease. YLL accounts for not only the number of deaths but also the age of death in relation to life expectancy to estimate better the mortality impact on population health due to a certain disease [7].

Currently, there is no scientific assessment available of the burden of COVID-19 on the Swedish population. Therefore, the aims of this study were to measure the disease burden of COVID-19 on the Swedish population during 2020–2021 overall and in different age groups and to compare it to the burden of other diseases.

## Methods

### Data sources and definition of COVID-19

This study used data from the nationwide multi-register observational study SCIFI-PEARL (Swedish COVID-19 Investigation for Future Insights – a Population Epidemiology Approach using Register Linkage). SCIFI-PEARL was designed in response to the COVID-19 pandemic, previously described in detail in a separate paper [8] and with further details given in the Supplemental Material (Appendix 1) of this paper.

In the current study, we included individuals with COVID-19, counting each individual once, based on the earliest registered COVID-19 date in any of the sources described (Table I). The index date was set to two days before that date, based on the assumption that symptoms started a few days before the individual had an indication of COVID-19 in our register data. Post-acute consequences of COVID-19 disease, referred to as post-COVID-19 condition (PCC) in this article, were defined according to the Global Burden of Disease (GBD) guidelines as prolonged symptoms such as tiredness, fatigue, pain all over the body, persisting after the acute infection and identified in our data set using the ICD code U09.9 for PCC (Table I) [9].

**Table I.** Health states definitions, disability weights and average durations in each health state for calculating DALY loss due to COVID-19 in Sweden.

Health state name	Health state definition	Disability weight	Average duration (source)
Infected	Individuals with positive test result for SARS-CoV-2 in SmiNet or indication of COVID-19 (ICD code (ICD-10-SE) U07.1 or U07.2) in the National Patient Register or the Cause-of-Death Register; index date was set to two days before the earliest captured registration for each individual [12]	Start of model	Start of model
Asymptomatic (no symptomatic period)	Infected individuals who have no registration of any health-care contact in the mild/moderate or later defined states	0	14 days
Mild/moderate	Infected individuals with registration of COVID-19 (ICD-code (ICD-10-SE) U07.1, U07.2) for outpatient visit in the National Patient Register (mild and moderate combined one health state)	0.051	14 days (Registry data)
Severe	Individuals with registration of hospitalisation with a COVID-19 code as primary diagnosis in the National Patient Register	0.133	10 days (Registry data)
Critical	Individuals who were transferred to or were directly admitted to an ICU as registered in the SIR	0.655	12 days (Registry data)
Post-COVID-19 condition	Individuals with registration of Long COVID code (ICD code U09.9) in the National Patient Register and or in one of the other data sets that are part of the study	0.219	57 days (Registry data)

SmiNet: national register of notifiable communicable diseases managed by the Public Health Agency of Sweden; YLL: years of life lost; YLD: years lived with disability; ICD code: code in the International Statistical Classification of Diseases and Related Health Problems, 10th revision; ICU: intensive care unit; SIR: Swedish Intensive Care Register.

### Calculation of YLL and YLD

A stochastic compartmental model was developed to estimate the YLL and YLD calculations. This model was guided by the European Burden of Disease Network and the European Centre for Disease Prevention and Control guidelines, described in detail elsewhere [9] The model and its different compartments are illustrated and explained in the Supplemental Material (Appendix 2) of this article. Microsoft Excel 365 was used for the preparation of charts and graphs, while the data set was analysed using the statistical package software Stata v17 (StataCorp, College Station, TX).

The DALYs were estimated by summing YLLs and YLDs [7]. The equations are as follows:

DALY=YLDs+YLLs

YLLs=the number of deaths (*n*)×the standard life expectancy at age of death

YLDs=the number of new cases of each health state (I)×a disability weight related to the health state (DW)×the average time a person stays in the health state

The number of deaths were extracted based on the relevant ICD code (ICD-10-SE) U07.1 or U07.2 denoting COVID-19 as an underlying or contributing cause of death in the Cause-of-Death Registry. To calculate YLL, mortality was counted as individuals who died at a particular age by each year and sex up to a theoretical maximum age of 110 years. Years lost due to death were calculated by multiplying the respective number of deaths in each age-by-sex category with the Sweden-specific life tables for estimated remaining life expectancy [10]. Pre-existing conditions were not considered in this calculation, similar to other studies [11] that are based on the GBD study which uses disease-independent age-specific residual life expectancy.

YLDs were calculated by multiplying the number of cases in each health state by the attributed disability weight suggested by the GBD guidelines [9], assuming an average duration in each health state. Some of the average durations could be calculated based on our data as average time spent in a certain health state. When this was not possible assumptions were based on recommendations by GBD [12]. Table I describes the health states used, with their definitions, disability weights and average durations, including sources for the latter. State transitions are illustrated in the Supplemental Material (Appendix 2).

### Uncertainty and sensitivity analysis

Sensitivity analyses were carried out to determine the impact of using different assumptions for some parameters [7].

The first parameter of interest was related to estimating death due to COVID-19. For the sensitivity analysis, we defined COVID-19 death as any death within 30 days of a positive test [13].

Another important factor influencing YLL is estimated life expectancy. In the main analysis, a Swedish national life table was used [10], while the aspirational life table recommended by the GBD guidelines was used for sensitivity analysis [12].

Finally, due to the lack of a clear definition and estimation of PCC, we tested different scenarios in terms of prevalence and duration of the health state, and we present the results of these scenario analyses. According to the GBD guidelines [9], when data about PCC are not available nationally, it could be assumed that 1/7 (13.3%) of the cohort enter the PCC state for an average of 28 days. Therefore, these prevalence and duration parameters were used in the sensitivity analysis, in addition to a scenario of doubling the local prevalence to account for potential underestimation.

### Comparison of leading causes of DALYs lost

The GBD has published and summarised the leading causes of DALYs lost worldwide, including Sweden, in their online database [14]. The latest published data are from 2019. We used the Swedish data to contextualise the burden of COVID-19 by comparing it to the burden of other communicable and non-communicable diseases reported for Sweden. For comparison, we used our results on COVID-19 burden for 2020 from the sensitivity analysis using aspirational life tables.

### Ethics

Ethical approval was obtained from the Swedish Ethical Review Authority (2020-01800), with several ethics amendments. The study population selection and pseudonymisation of the data are managed by Statistics Sweden and the National Board of Health and Welfare. Researchers do not have access to the code key for identifying study participants.

## Results

### General impact on the Swedish population

The number of COVID-19 patients in different states detected between March 2020 and October 2021 is summarised in Table II. Patients in a severe state were hospitalised for an average of 10 days, which led to a loss of 216 YLD (19.6% of the total YLD), while patients in the critically ill state stayed in the intensive care unit (ICU) for an average of 12 days (critical state), hence contributing to 169 YLD (15.4% of the total YLD). In the cohort, 1.07% experienced post-COVID-19 consequences, and we estimated an average of 57 days in this health state. As a result, PCC contributed to 46% of the total YLD in the cohort.

**Table II.** Burden of COVID-19 in the Swedish population 2020–2021 using SCIFI-PEARL data.

	Number of Cases	YLD	YLL	DALYs
Morbidity				
Mild/moderate	106,902	209		
Severe	59,415	216		
Critical	7863	169		
Long COVID	14,766	505		
Mortality	16,166		151,778	
Total burden of disease		1099	151,778	152,877

SCIFI-PEARL: Swedish COVID-19 Investigation for Future Insights – a Population Epidemiology Approach using Register Linkage; YLD: years of healthy life lost due to disability; YLL: years of life lost; DALYs: disability-adjusted life years.

The total burden of COVID-19 during the study period was 152,877 DALYs (Table II). Premature mortality contributed 99.3% of the DALYs burden in Sweden, with 58.5% of YLL burden occurring during 2020, leading to an estimated burden of 1418 DALYs loss per 100,000 people in Sweden in that year among a national population of 10.35 million [15].

### Demographic parameters

**Figure 1.**
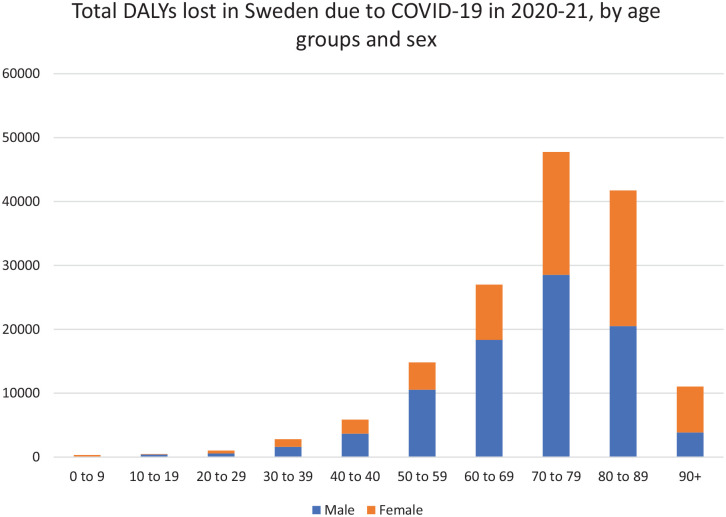
Distribution of the burden of COVID-19 (DALYs lost) in the Swedish population, by age groups and sex. DALY: disability-adjusted life year.

More than half (57.6%) of the burden was attributed to men, who had higher number of DALYs in all age groups except ⩾90 years (Figure 1). The DALY burden was highest among the elderly, with 83.4% of the total loss of DALYs estimated among individuals ⩾60 years: Of the total burden, 30% and 31% were estimated in the age groups 70–79 and 80–89 years old, respectively (Figure 1).

### Sensitivity analyses

When we estimated YLL using 30-day mortality after a positive SARS-CoV-2 test instead of cause-specific COVID-19 mortality, total DALYs were 4% lower than in the baseline analysis. The decrease occurred among women, where estimated DALYs lost decreased from 64,255 to 58,023, while the burden among men slightly increased from 87,523 to 87,861. Consequently, men carried 60% of the total burden in this analysis. The highest loss of DALYs remained among the age groups 70–79 and 80–89 years (31% and 27% of the total burden, respectively).

Using the GBD life table as a life expectancy standard to calculate YLL led to higher estimates for both men and women in all age groups. The total YLL in the cohort increased by 61%, from 151,778 to 244,511. As result, the total DALY burden during the study period increased to 245,610, of which 146,878 occurred in 2020, corresponding to a rate of 1419 DALYs per 100,000 individuals of the Swedish population.

When increasing the PCC prevalence in the cohort to 2.14%, 4.28% and 13.3%, the total DALY burden increased by 0.3%, 1% and 4.1%, respectively (Table III). When changing the duration of PCC to 28 days, according to GBD guidelines, the total YLD and therefore total DALYs decreased in all the tested scenarios.

**Table III.** Sensitivity analyses related to post-COVID-19 condition in the calculation of YLDs lost for the Swedish population during 2020 to October 2021.

Scenario (prevalence; duration)	Total DALYs	Change (%)
1.07%; 57 days (baseline analysis)	152,877	
2.14%; 57 days	153,382	0.33
4.28%; 57 days	154,392	0.99
13.3%; 57 days	159,154	4.11
2.14%; 28 days	153,125	−0.16
4.28%; 28 days	154,135	−0.82
13.3%; 28 days	158,897	−3.94

### Comparison of leading causes of DALYs lost

**Figure 2.**
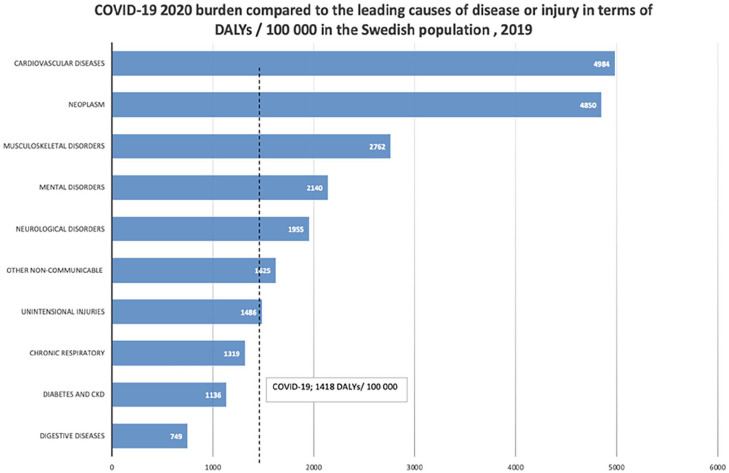
Estimates of COVID-19-related DALYs (2020) and pre-pandemic leading causes of disease/injury (2019) in Sweden. CKD: chronic kidney disease.

The 2020 COVID-19 burden was compared to the burden related to the top leading causes of disease and injuries in Sweden in 2019 based on the latest reported GBD figures for loss of DALYs in the country (Figure 2) [14]. Although COVID-19 is the leading communicable disease, as expected, it ranks only eighth on the list of all causes, preceded by a range of non-communicable diseases, including cardiovascular diseases, neoplasms and musculoskeletal disorders.

## Discussion

During the first 20 months of the COVID-19 pandemic, a total of 152,877 DALYs were estimated to have been lost due to the disease in the Swedish population. The disease burden was mainly due to premature mortality rather than morbidity. Although COVID-19 was the major cause of morbidity and mortality due to infectious disease during the recent period, it impacted the Swedish population’s health less in terms of loss of DALYs than several non-communicable diseases, such as cardiovascular diseases and cancers, which were the two leading causes of death and loss of DALYs in 2020 [16].

The results of our study are in line with other international observations in terms of the demographic distribution of COVID-19 burden. The majority of deaths occurred among the elderly, which could be attributed to the fact that the elderly had a higher prevalence of risk factors for severe COVID-19 disease [17]. Previous research in Sweden has highlighted that unmarried elderly individuals are at a particularly high risk of dying from COVID-19, potentially due to their need to rely on external help or living in a care home [18]. Residing in an elderly care home in Sweden was associated with increased mortality due to COVID-19. This increased risk could be potentially explained by the exposure to visitors and care workers and by the poor underlying health among the elderly care home residents [19]. Therefore, our findings, along with other research, highlight again the need to consider strategies to protect this vulnerable group, with emphasis on elderly care home residents in Sweden, while focusing on prevention and efficient management of infectious outbreaks. This is even more important, since although most deaths from COVID-19 occurred in the elderly where the remaining life expectancy is more limited, the total sum of YLL is substantial.

In our analysis, Swedish men experienced a higher loss of DALYs than women did. In terms of mortality, this could be explained by two factors: first, the higher number of men dying than women in all the age groups included in the analysis, and second, the distribution of deaths, which was skewed more towards younger age for men compared to women, leading to higher YLL in men. These findings are in line with current evidence that male gender is associated with a higher risk of mortality due to COVID-19 [20,21]. In terms of demographic distribution of COVID-19 burden, Sweden followed the general pattern reported in most countries worldwide.

Due to the similar methodology that we used in our sensitivity analysis by using the GBD life tables to estimate YLL, we were also able compare findings from our study directly with other studies that have estimated the burden of COVID-19 on population health with such methodology. Four studies were identified as suitable for comparison: from Germany, Malta, the Netherlands and Scotland [11,22–24]. In all of these countries, YLL dominated the total DALYs: Germany and the Netherlands had the same figure as Sweden (around 99.3%) [11,24], with a low YLD contribution of 0.7%. The YLD contribution was 2% in Scotland [24] and was highest (5%) in Malta [22]. The estimated DALY disease burden in the Swedish population in 2020 in this analysis was 1418 DALYs per 100,000 people, which was lower than in the Netherlands and Scotland (1570 and 1770 per 100,000, respectively). The burden was significantly lower in Germany than in Sweden in 2020 (542/100,000, using the GBD life table). According to the researchers, this might be explained by the way of reporting death due to COVID-19 used in that study, which may have underestimated the real fatality of the disease, or it could be reflective of better pandemic control strategies adopted in the country [11].

However, compared to other Nordic countries, Sweden had a higher rate of COVID-19 hospitalisations and mortality, which engendered some criticism against the ‘relaxed’ response that Sweden adopted during the pandemic. Therefore, the DALYs burden is expected to be higher than for neighbouring countries such as Denmark, Finland, Norway and Iceland [25]. To date, however, there are no published studies about the DALYs burden of COVID-19 in these countries, and therefore no firm conclusions or comparisons can be made. In particular, criticism targeted the Public Health Agency overall approach, as well as the measures taken by the municipalities to protect residents in elderly care home [26]. On the other hand, the health-care system in Sweden was not overwhelmed in the same way as it was in some other European countries, and the sustained relatively trustful relationship between the government and the citizens in Sweden was perhaps an illustration of a sustainable approach where the main responsibilities were devolved to the individuals [27]. It is unclear whether a higher level of population-level restrictions would have led to a lower loss of DALYs in the Swedish setting.

## Methodological considerations

### YLD calculation

Although the YLDs ultimately contributed to a minimal proportion of the total DALYs, calculation of YLD raised many methodological challenges.

Due to the limitations of a register-based study, mild and moderate COVID-19 had to be classified as one health state based on a registration of COVID-19 (ICD code U07.1, U07.2) in outpatient specialist care in the National Patient Register. Individuals in the mild/moderate state were assumed to have experienced sufficient symptoms to seek health care in a specialist setting. Individuals who were classified as asymptomatic were those who were not registered as having COVID-19 in the National Patient Register. They were considered asymptomatic under the assumption that they would otherwise have sought medical help. However, many of them likely had symptoms managed through self-care and visits to pharmacies or health centres from which data were not available. The proportion of people with a positive test who had symptoms was likely high, since there was no mass screening of asymptomatic individuals in Sweden, and there were very few situations that required mandatory testing. Nonetheless, for people residing in Sweden, a strong recommendation to get tested when suspected symptoms occurred (or when infection through contact with other infected or sick individuals was likely to have occurred), and to self-isolate if positive, was issued by the Public Health Authority. However, in the first months of the pandemic, test capacity was limited, and only symptomatic risk groups were recommended testing, while others were asked to stay home and not seek health care for mild symptoms [28,29]. We would argue that the testing and screening strategies in Sweden led to a potential underestimation of patients with especially asymptomatic and mild/moderate symptoms and that the actual burden for these health states was higher in reality. However, the asymptomatic health state, for which the GBD does not yet have a suggested disability factor, is likely to be mild and have short duration, and inclusion of this health state would probably not have a major impact on the total YLD or DALY.

As the majority of YLD was contributed by PCC, it is important to highlight the challenge of defining PCC and the period of this health state. These challenges, in addition to the need to generate better disability weights for this health state, remain areas of concern, as has repeatedly been pointed out in previous publications on this topic. To tackle this uncertainty, sensitivity analyses were conducted varying the main parameters (duration and prevalence of PCC), and our total DALY results were robust across these analyses. However, there remains a need for better research to understand the impact of PCC in Sweden as well as other international contexts for better estimation of DALYs.

### YLL calculation

The high contribution of YLL to the COVID-19 disease burden is grounded in the relatively high mortality rates due to the disease, especially in the early stages of the pandemic, affecting individuals of all ages but particularly the elderly. However, the extent of the estimated YLL is sensitive to the definition of COVID-19-related mortality. We conducted analyses based on two common definitions of COVID-19 mortality, and our results were robust across these analyses.

Another methodological discussion concerning YLL is whether the choice of mortality risks (remaining life expectancy) is best based on local (e.g. national) current life tables or on a so-called aspirational life table. The advantages and disadvantages of each choice have been discussed in the literature [30]. A strength of this study is that we conducted the analysis and presented results using both alternatives, which allows for comparison with estimates of loss of DALYs from other countries that have used either method.

Finally, mortality is highest in the elderly with underlying health conditions. Our analysis did not account for shorter remaining life expectancy in this group than that related to age, which most likely has resulted in an overestimation of YLL. However, our main approach used current Swedish life tables, which should result in less overestimation. In the sensitivity analysis using the recommended GBD approach with aspirational life tables, which allows for international comparisons, the likely overestimation is higher.

### Potential factors related to DALYs burden

Socio-economic characteristics such as income, profession and legal status in Sweden could be potential factors influencing the burden of DALYs in Sweden. However, our analysis did not adjust for such factors which is a limitation of this study. Another important aspect is the distribution of the DALYs burden across different cohorts in terms of their vaccination status as well as the distribution during different phases of the pandemic depending on the COVID-19 vaccination program coverage. Although these vaccination parameters were not included in this analysis, they could potentially be assessed in the future for a better understanding of the burden of COVID-19.

## Conclusions

The burden of COVID-19 in Sweden is comparable to some other European countries in terms of the rate of DALYs per 100,000 individuals and demographic distribution of DALYs lost. The disease burden was concentrated mainly among the elderly and is related to mortality rather than morbidity. Despite the fact that the COVID-19 burden was mainly due to premature mortality in the older age groups, more research is still needed regarding PCC disability to understand better the morbidity that may be related to this disease. Emphasising both YLL and YDL components of DALYs supports a better understanding of the effectiveness of past policies and their impact on population health and provides insights for future pandemics and control of disease outbreaks.

## Supplemental Material

sj-docx-1-sjp-10.1177_14034948231160616 – Supplemental material for The burden of disease due to COVID-19 in Sweden 2020–2021: A disability-adjusted life years (DALYs) study Click here for additional data file. Supplemental material, sj-docx-1-sjp-10.1177_14034948231160616 for The burden of disease due to COVID-19 in Sweden 2020–2021: A disability-adjusted life years (DALYs) study by Jad Shedrawy, Patricia Ernst, Knut Lönnroth and Fredrik Nyberg in Scandinavian Journal of Public Health
