# Supplementary material for: The burden of disease due to COVID-19 in Sweden 2020–2021: A disability-adjusted life years (DALYs) study
Source: Scand J Public Health. 2023 Mar 20;51(5):673–81. doi: 10.1177/14034948231160616 (PMC10033504; doi:10.1177/14034948231160616)
Supplement: sj-docx-1-sjp-10.1177_14034948231160616 – Supplemental material for The burden of disease due to COVID-19 in Sweden 2020–2021: A disability-adjusted life years (DALYs) study [file sj-docx-1-sjp-10.1177_14034948231160616.docx]

**Electronic appendix**

to

**The burden of disease due to COVID-19 in Sweden 2020-21: a disability-adjusted life years (DALYs) study**

Jad Shedrawy^1*^, Patricia Ernst^2^, Knut Lönnroth^1 3^, Fredrik Nyberg^2^

1. Department of Global Public Health, Karolinska Institutet, Stockholm, Sweden.
2. School of Public Health and Community Medicine, Institute of Medicine, Sahlgrenska Academy, University of Gothenburg, Gothenburg, Sweden
3. Centre for Epidemiology and Community Medicine, Stockholm County Council, Stockholm, Sweden.

**eAppendix 1 - Description of the SCIFI-PEARL database, and data items from the database of relevance for the current analyses of DALYs**

The SCIFI-PEARL (Swedish Covid-19 Investigation for Future Insights – a Population Epidemiology Approach using Register Linkage) project was originally conceived with an overall case-cohort design that included all COVID-19 cases identified prospectively and a population random sample of approximately 1 million individuals, as described in detail elsewhere (E1). At present, it has been reshaped and expanded to a true national cohort design and includes all individuals in the Swedish population on 1 January 2015 or any time thereafter, and COVID-19 cases are identified prospectively among these in the study database using extensive data from different sources.

For the purposes of the current study of DALYs due to COVID-19, the SCIFI-PEARL database thus includes all individuals with a positive SARS-CoV-2 polymerase chain reactions (PCR) test, COVID-19 diagnosis or registration for a healthcare visit or hospitalisation, or COVID-19 as cause of death, identified from a large number of national and regional databases during the pandemic. Persons with a positive SARS-CoV-2 test were identified from the Public Health Agency of Sweden (Folkhälsomyndigheten), based on mandated notifications through SmiNet (E2), the national registry of notifiable communicable disease. COVID-19 patients with or without a positive test were also identified through registries capturing registered encounters with the healthcare system where relevant COVID-19 International Classification of Diseases, rev. 10, Swedish version (ICD-10-SE) codes (U07.1 or U07.2) had been entered. On national level, such information was extracted from the National Patient Register (NPR) for outpatient and hospitalized inpatient specialist care (E3). Patients treated in intensive care units were captured from the Swedish Intensive Care Register. Mortality due to COVID-19 was obtained from the National Cause-of-Death Register (E4), for deaths with the mentioned ICD codes as underlying or contributing cause of death. The appropriate dates of testing, healthcare visits, hospital or intensive care unit (ICU) admission and/or death were obtained from these registers.

**References**

E1. Nyberg F, Franzén S, Lindh M, Vanfleteren L, Hammar N, Wettermark B, Sundström J, Santosa A, Björck S, Gisslén M. Swedish Covid-19 Investigation for Future Insights - A Population Epidemiology Approach Using Register Linkage (SCIFI-PEARL). Clin Epidemiol. 2021 Jul 30;13:649-659.

E2. Rolfhamre P, Janson A, Arneborn M, Ekdahl K. SmiNet-2: Description of an internet-based surveillance system for communicable diseases in Sweden. Euro Surveill. 2006;11(5):pii=626.

E3. Ludvigsson JF, Andersson E, Ekbom A, Feychting M, Kim JL, Reuterwall C, Heurgren M, Olausson PO. External review and validation of the Swedish national inpatient register. BMC Public Health. 2011 Jun 9;11:450.

E4. Brooke HL, Talbäck M, Hörnblad J, Johansson LA, Ludvigsson JF, Druid H, Feychting M, Ljung R. The Swedish cause of death register. Eur J Epidemiol. 2017 Sep;32(9):765-773.

**eAppendix 2- Description of the model**

The model is a stochastic compartmental model where patients move between 8 different health states (Figure e1). All patients enter the model in the health state “Infected”. Following that, patients move to one of 3 health states depending on whether they are registered dead or survived as "Asymptomatic" individuals or as "Symptomatic".

Individuals who were only observed in the “Asymptomatic” health state did not contribute to the burden of the disease and it was assumed that this cohort fully recovers and move to the health state “recovered”, excluding the possibility of post-COVID in the current analysis model, due to the lack of such data in this cohort.

Individuals within the “Dead” health state contributed only to the mortality burden of the disease, which has been modelled using different approaches, as detailed in the main text of the paper.

The "Symptomatic" health state has been modelled as a sequence of 3 stages “Symptomatic mild/moderate”, “Symptomatic severe” and “Symptomatic critical”. This sequence determined the calculations of morbidity due to acute COVID-19 in the cohort. An assumption has been made that all patients whose first record is a hospital/ICU admission would have passed through the “mild/moderate” health state 14 days prior to their admission. Symptomatic patients at any stage of the sequence are assumed at risk of developing post-COVID condition and could move to the “Post-COVID” health state. Since all individuals who were observed to have died were assessed in the mortality burden part of the model, all "Symptomatic" patients in the model, with or without post-COVID condition, eventually recover and move to the health state “Recovered”.


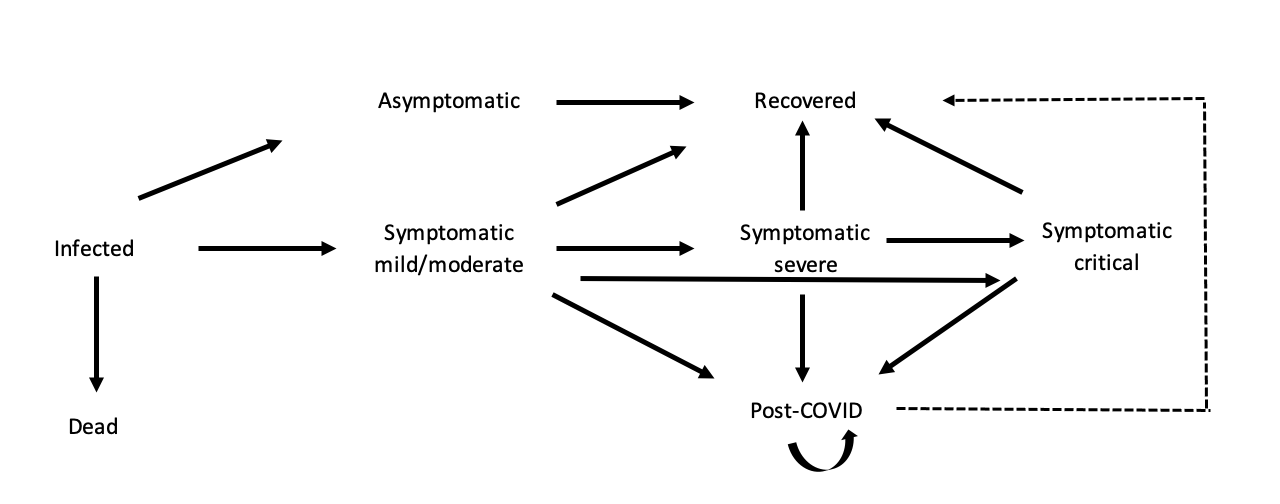


Figure e1. Structure of the model representing the different health state of COVID-19 patient cohort
